# 

**Published:** 1935

**Authors:** 


					Vol. LH. No. 196.
?
- if
..>? ?;?"?-? nr?f\
.Mmmm HM- H ?
SUMMER, 1935.
?" Tc. Hi
mi. ri . ^ _ i
The Bristol
Medico-Chirurgical Journal
,
PUBLISHED UNDER THE A.U8PI0E8 OF
" ? - * ?" --y
THE BRISTOL MEDICO-CHIRURGICAL SOCIETY.
Editor: J. A. NIXON, C.M.G., M.D., F.R.C.P.
WITH WHOM ARE ASSOCIATED
/ '? ? r?
E. W. HEY GROVES, D.Sc., M.S., F.R.O.S. :' ^|1
A. E. ILES, O.B.E., M.B., B.S., F.R.C.S.
A. RENDLE SHORT, M.D., B.S., B.So., F.R.C.8.
E. WATSON-WILLIAMS, M.C., Ch.M., F.R.O.S., Assistant Editor.
Editorial Secretary: A. '|U FLEMMING, M.B., Ch.B.
I . ,H?w JjjP
. . ? - " f ? > ' ;i?:p m ? ?'
?W5Pr- ? V*. "- 'V ' <**h
; I w. '
.ONDON : BJBLjm,; ?
Hohsk,
Price Three Shillings net.
* fWTSP m g> r A-r ?-? -

				

